# Construction of a SNP-Based Genetic Map Using SLAF-Seq and QTL Analysis of Morphological Traits in Eggplant

**DOI:** 10.3389/fgene.2020.00178

**Published:** 2020-03-11

**Authors:** Qingzhen Wei, Wuhong Wang, Tianhua Hu, Haijiao Hu, Jinglei Wang, Chonglai Bao

**Affiliations:** Institute of Vegetables, Zhejiang Academy of Agricultural Sciences, Hangzhou, China

**Keywords:** SLAF sequencing, SNP markers, eggplant, genetic linkage map, QTL analysis

## Abstract

Eggplant (*Solanum melongena*; 2*n* = 24) is an economically important fruit crop of the family Solanaceae that was domesticated in India and Southeast Asia. Construction of a high-resolution genetic map and map-based gene mining in eggplant have lagged behind other crops within the family such as tomato and potato. In this study, we conducted high-throughput single nucleotide polymorphism (SNP) discovery in the eggplant genome using specific length amplified fragment (SLAF) sequencing and constructed a high-density genetic map for the quantitative trait locus (QTL) analysis of multiple traits. An interspecific F_2_ population of 121 individuals was developed from the cross between cultivated eggplant “1836” and the wild relative *S. linnaeanum* “1809.” Genomic DNA extracted from parental lines and the F_2_ population was subjected to high-throughput SLAF sequencing. A total of 111.74 Gb of data and 487.53 million pair-end reads were generated. A high-resolution genetic map containing 2,122 SNP markers and 12 linkage groups was developed for eggplant, which spanned 1530.75 cM, with an average distance of 0.72 cM between adjacent markers. A total of 19 QTLs were detected for stem height and fruit and leaf morphology traits of eggplant, explaining 4.08–55.23% of the phenotypic variance. These QTLs were distributed on nine linkage groups (LGs), but not on LG2, 4, and 9. The number of SNPs ranged from 2 to 11 within each QTL, and the genetic interval varied from 0.15 to 10.53 cM. Overall, the results establish a foundation for the fine mapping of complex QTLs, candidate gene identification, and marker-assisted selection of favorable alleles in eggplant breeding.

## Introduction

Eggplant (*Solanum melongena* L., 2*n* = 24) is an important vegetable crop cultivated worldwide that belongs to the large family Solanaceae. The total global production of eggplants accounted for ∼52.3 million tons in 2017 (FAOSTAT 2017^1^). In contrast to many New World-originating vegetables within the family, such as tomato, potato, and pepper, eggplant most likely originated in the Old World (Daunay et al., 2001). Eggplants exhibit extensive variations in leaf morphology, fruit size and shape, and plant architecture among cultivated varieties and wild relatives. However, map-based gene cloning, as well as the understanding of the molecular and genetic mechanisms underlying horticulturally important traits in eggplant, have largely lagged behind compared to other vegetable crops (i.e., tomato and cucumber) due to the limited number of molecular markers and relatively low density of genetic maps.

In the past decades, a number of eggplant linkage maps have been constructed with both dominant and co-dominant DNA markers, which have been used to map disease resistance and plant morphology traits (Nunome et al., 2001, 2003, 2009; Wu et al., 2009; Barchi et al., 2010, 2012, 2018; Lebeau et al., 2013; Frary et al., 2014; Miyatake et al., 2016). The first eggplant genetic map was constructed by Nunome et al. (2001) using dominant markers [randomly amplified polymorphic DNA (RAPD) and amplified fragment length polymorphism (AFLP) markers] and an intra-specific EWF_2_ population; several markers were associated with fruit shape and color. However, the map contained only 181 markers and covered 21 linkage groups. Subsequently, Nunome et al. (2003, 2009) developed ∼1000 simple sequence repeat (SSR) markers with which two genetic maps were constructed using the same F_2_ population. Although these linkage maps are saturated, they have been useful as important co-dominant marker resources for marker-assisted selection in eggplant breeding. Miyatake et al. (2012) constructed two linkage maps containing SSRs and single nucletide polymorphisms (SNPs) and identified linked markers for parthenocarpy traits using NAF2 and ALF2 populations. To date, the most saturated genetic map was constructed by integrating the SSRs and SNPs with another two F_2_ populations (LWF2 and EWF2); in total, 1,745 loci were mapped in the integrated map (Fukuoka et al., 2012; Hirakawa et al., 2014). This map was used to facilitate the assembly of eggplant draft genome sequences.

In parallel with the advances in the genetic linkage maps, the identification of quantitative trait locus (QTLs) associated with agronomic traits has been greatly promoted in eggplant. Doganlar et al. (2002a) constructed a molecular linkage map with tomato restriction fragment length polymorphism (RFLPs) and an interspecific F_2_ population (58 individuals). A total of 125 significant QTLs associated with domestication and morphological traits were detected using the interspecific linkage map in the same segregation population (Doganlar et al., 2002b; Frary et al., 2003; Wu et al., 2009). Sunseri et al. (2003) developed an RAPD-AFLP map with 273 markers and identified molecular markers linked with *Verticillium* wilt. Barchi et al. (2010, 2012) identified a number of QTLs controlling anthocyanin pigmentation using an intra-specific F_2_ population derived from 305E40 × 67/3 and a 238-loci linkage map. With the same linkage map and F_2_ population, QTLs related to total and early yield, fruit traits (e.g., weight, length, diameter, and shape), prickliness traits, and fruit metabolic content were mapped, and, for each trait, at least one major QTL was identified (Portis et al., 2014; Toppino et al., 2016). Major and minor QTLs affecting resistance to *Fusarium* and *Verticillium* in the intraspecific 305E40 × 67/3 map were also detected, and putative orthologous genes from tomato were identified (Barchi et al., 2018). In addition, genome-wide association analysis (GWAS) also plays an important role in genetic mapping of relavent traits in eggplant. Using this approach, Cericola et al. (2014) and Portis et al. (2015) identified a number of phenotype/genotype associations for key breeding fruit and plant traits with 191 mixed eggplant accessions. Nonetheless, despite the progress in QTL detection, most of the traits were analyzed in intraspecific populations, and the linkage maps used in genetic mapping are still less saturated.

Large-scale DNA marker development and the construction of a high-resolution linkage map in eggplant would provide fundamental tools for map-based gene mining. Advances in next-generation sequencing (NGS) technologies provide an excellent opportunity to develop abundant SNP markers for linkage map construction. Restriction-site associated DNA sequencing (RAD-seq) and 2b-RAD are both useful tools for SNP discovery; they reduce genome complexity by sequencing only DNA fragments with restriction sites despite fragment length (Miller et al., 2007; Baird et al., 2008; Ogden, 2011; Wang et al., 2012). Specific length amplified fragment (SLAF) sequencing (SLAF-seq) is an improved reduced representation library (RRL) sequencing strategy that brings down the cost through genome reduction. SLAF-seq has been proved an effective method for *de novo* SNP discovery and high-throughput genotyping and has wide applications in genetic map construction in sesame (Zhang et al., 2013), kiwifruit (Huang et al., 2013), soybean (Qi et al., 2014; Zhang et al., 2018), cucumber (Wei et al., 2014; Zhu et al., 2016), peanut (Hu et al., 2018), sweet osmanthus (He et al., 2017), and cotton (Zhang et al., 2016; Ali et al., 2018). In the present study, we developed an interspecific F_2_ population containing 121 individuals from a cross between an eggplant cultivar and the wild relative *S. linnaeanum*. Using the F_2_ population and SLAF-seq technology, a high-density genetic map with 2,122 SNP markers was constructed. Importantly, 19 QTLs were detected for plant architecture-, fruit-, and leaf-related traits.

## Materials and Methods

### Plant Materials and Phenotyping

**FIGURE 1:**
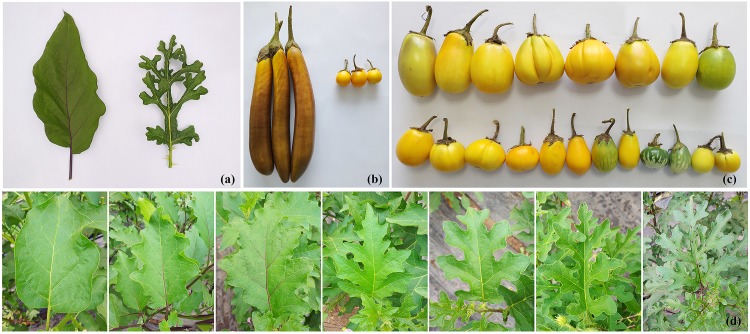
Fruit and leaf morphology of the two eggplant parental lines and the F_2_ population. **(a)** Leaves of *Solanum linnaeanum* “1809” (right) and *S. melongena* “1836” (left); **(b)** mature fruits of “1809” (right) and “1836” (left); **(c)** mature fruits of the representative F_2_ individuals; **(d)** leaves of the representative F_2_ individuals.

The cultivated eggplant *S. melongena* “1836” and its wild relative *S. linnaeanum* “1809” were used as male and female parents, respectively. “1836” is an inbred line with long, purple fruits and few prickles, whereas “1809” is prickly and produces small, round, green, and striped fruit (Figure 1). In this study, an interspecific F_2_ population containing 121 individuals was generated from a cross between “1809” and “1836,” which was then used as the mapping population. The parents and the F_2_ population were grown in spring 2018 in the greenhouses at Qiaosi experiment field of Zhejinag Academy of Agricultural Sciences, Hangzhou, China, with plant spacing of 60 cm, row spacing of 1.2 m, and ridge cultivation.

We collected data on the following eight traits from the F_2_ plants: main stem height (MSH), fruit length (FL), fruit diameter (maximum diameter; FD), fruit shape (FL/FD; FS), leaf lobing (LLOB), leaf prickle number (LPN), leaf prickle color (LPC), and vein color (VC). The height of the main stem (in cm) is the length from above the ground to the pseudobinary branch, which was determined in Qiaosi in mid-July. Fruit character measurements (in mm) were taken from mature fruits at the beginning of July. For fruit-related traits, three fruits were measured for each F_2_ plant, and the average value was used as the final fruit trait value for QTL mapping. Plants with fewer than three fruits were considered as N/A, since some F_2_ individuals could not produce fruits by self-pollination. Leaf lobing was assessed on a 1–7 scale (1 for no fissures at leaf margin; 7 for leaf margin deeply cleft); leaf prickle number was assessed in the same way (1 for no prickles; 7 for many prickles). Leaf prickle color was assessed on a 1-4 scale (light green, green-purple, green-brown, and dark purple). Leaf vein color was scored on a 1 to 3 scale (green, green and purple, and purple). All leaf morphology traits were measured at the beginning of July in Qiaosi.

### DNA Extraction

Young, healthy leaves from both eggplant parents and the 121 F_2_ individuals were collected, frozen in liquid nitrogen, and then transferred to a −80°C freezer. Total genomic DNA was extracted from each leaf sample according to the cetyltrimethyl ammonium bromide (CTAB) method (Murray and Thompson, 1980). DNA concentration and quality were examined by electrophoresis on 1% agarose gels using a standard lambda DNA and an ND-1000 spectrophotometer (NanoDrop, Wilmington, DE, United States).

### SLAF Library Construction and Sequencing

SLAF-seq was used to genotype the two parents and 121 F_2_ individuals according to previously described procedures (Sun et al., 2013; Wei et al., 2014) with some modifications. The predicted electronic enzymatic digestion was performed using the eggplant genome sequence (Hirakawa et al., 2014) as a reference genome, and a combination of two endonucleases (*Rsa*I and *Hae*III) was selected. The digested fragment sequences with lengths of 414–464 bp were defined as a SLAF Label. The PCR products were purified using a Gel Extraction Kit (Qiagen), and then the gel-purified products were sequenced on an Illumina HiSeq 2500 system (Illumina, Inc., San Diego, CA, United States) according to the manufacturer’s recommendations. The ratio of raw high-quality reads with quality scores greater than Q30 (a quality score of 30 indicates a 0.1% chance of obtaining an error, and thus 99.9% confidence) and the guanine-cytosine (GC) content were calculated for quality control.

### SNP Discovery and Genotyping

SLAF-seq data were assessed using the software developed by Sun et al. (2013), and SNP markers were identified and genotyped according to the procedures described by Sun et al. (2013) and Wei et al. (2014) with modifications. Sequences with over 95% similarity were considered as one SLAF locus. The clean reads were mapped to the eggplant reference genome (Barchi et al., 2019), and the GATK software kit was used to detect SNPs between two parents and F_2_ offspring. For the detailed process, see GATK’s official website, https://www.broadinstitute.org/gatk/guide/best-practices.php (McKenna et al., 2010). SLAFs with two to four alleles were identified as polymorphic and considered potential markers. All polymorphism SLAFs were genotyped with consistency in the parental and offspring SNP loci. The polymorphic SLAF markers were sorted into eight segregation patterns as follows: ab × cd, ef × eg, hk × hk, lm × ll, nn × np, aa × bb, ab × cc, and cc × ab. Since the mapping populations were derived from two homozygous eggplant parents with a genotype of aa or bb, only the SLAF markers with the segregation pattern aa × bb were used.

### Analysis of Segregation Distortion and Marker Filtering

To guarantee the quality of markers on the linkage map, we performed a strict marker filtering process according to the sequencing depth of the parents, marker completeness (coverage), and *P*-value of segregation distortion. Only SNPs with parental sequence depths of more than 10 × were retained, the complete degree parameters were set as 70 and 75%, and the *P*-values were set at less than 0.05, 0.01, and 0.001. The parameters were arranged in seven combinations to filter and analyze the markers.

According to the analysis of segregation distortion, we used two rules to filter the polymorphic markers in order to ensure the quality of the genetic map and the uniform distribution of markers. For linkage groups (LGs) 1, 3, 4, 6, 7, 8, 9, and 11, the following filtering principles were employed. (1) Filtering by sequence depth: remove SNPs with parental sequence depths of less than 10 ×. (2) Filtering by sequence completeness: remove SNPs with complete degree below 70%. (3) Filtering by segregation distortion: remove SNPs with serious segregation distortion (*P*-value < 0.01). Two additional filtering principles used for LGs 2, 5, 10, and 12 were as follows. (1) Filtering by sequence depth: remove SNPs with parental sequence depths of less than 12 ×. (2) Filtering by sequence completeness: remove SNPs with complete degree below 90%. According to these parameters, only high-quality SNPs were selected as potential markers.

### Linkage Map Construction

The high-quality SNP markers were arranged and genotyping errors were corrected by the HighMap strategy and the SMOOTH algorithm, respectively (van Os et al., 2005; Liu et al., 2014). The k-nearest neighbor algorithm was used to manage missing genotypes (Huang et al., 2011). Then, the genetic linkage map was constructed by Joinmap v5.0^2^, according to the regression algorithm method. The Kosambi mapping function was used to estimate the genetic distance (cM) of adjacent markers.

### QTL Analysis

Quantitative trait locus analysis was performed with R/qtl^3^. Composite interval mapping (CIM) was used to identify QTLs. The logarithm of odds (LOD) threshold was used to evaluate the statistical significance of each QTL and was set by 1000 permutations test (PT). To ensure that both major and minor effect QTLs could be identified, different LOD scores were adopted. Firstly, a LOD threshold corresponding to 0.99 confidence was considered, and if there was no mapping interval, a LOD threshold corresponding to 0.95 confidence was used; if there was still no positioning interval, the threshold value of 0.90 confidence was considered. Finally, if there was still no QTL interval detected, the PT result was not used, and the threshold was manually lowered to 3.0, 2.5, and 2.0. QTLs were named according to their linkage group locations and trait names. For example, *fl1.1* referred to the first QTL for fruit length on eggplant LG 1.

## Results

### SLAF-Sequencing and SNP Marker Analysis

After SLAF library construction and high-throughput sequencing, a total of 111.74 GB of data comprising 487.53 M paired-end reads was generated. Among these reads, 94.04% achieved or exceeded a quality score of 30 (Q30, indicating a 0.1% chance of an error, and 99.9% confidence) and the guanine-cytosine (GC) content was 38.83%. The average sequencing depth was 19.48 × for the female parent “1809,” 19.46 × for the male parent “1836,” and 10.29 × for F_2_ progeny. GATK software was used to develop parent and offspring SNP markers combined with the eggplant reference genome. In total, 13,455,526 SNP markers were developed, in which 11,386,169 SNPs were successfully encoded and grouped into eight segregation patterns (ab × cd, ef × eg, hk × hk, lm × ll, nn × np, aa × bb, ab × cc, and cc × ab; Supplementary Table S1). 9,971,182 SNPs fell into the segregation pattern of aa × bb, accounting for 74.10% of the total developed markers, which were used for further analysis (Supplementary Table S1).

### Segregation Distortion

According to the three parameters, i.e., parent sequencing depth, coverage degree, and *P*-value of segregation distortion, we calculated clustering results under seven parameters (Table 1), and the remaining number of markers was between 10,773 and 64,320. When sequencing depth and coverage were the same, marker number tended to increase as the *P*-value decreased. We found that LG2, 5, 10, and 12 had significantly lower marker numbers after segregation distortion filtering, regardless of which *P*-value was used. LG2 had the lowest number of SNPs, which ranged between 26 and 46 under different parameter combinations. The marker number on LG5 varied from 80 to 588; when sequencing depth was set > 10 × and coverage degree was 75%, 80 SNPs were retained with *P*-values < 0.05, whereas 577 SNPs were retained with *P*-values < 0.001; thus most markers on LG5 were segregation distorted. Similar results were also observed for LG10 and LG12 (Table 1). The marker numbers on the four linkage groups were slightly improved after decreasing sequencing depth, coverage degree, and *P*-value. However, the four linkage groups had a significantly higher number of markers if not selected for segregation distortion. Based on these results, we adopted two principles in the marker filter process to ensure both marker number and quality (See section “Materials and Methods”).

**TABLE 1:** Number of SNP markers retained using different filtering parameters in 12 eggplant linkage groups.

Linkage group ID	Sequence Depth_Coverage_*P* value							
	
	10_0.7_0.05	10_0.7_0.01	10_0.7_0.001	10_0.75_0.05	10_0.75_0.01	10_0.75_0.001	10_0.75	12_0.1
E01	93	466	2339	93	465	2334	7010	2512
E02	26	32	46	26	32	46	5751	2186
E03	320	913	2300	320	913	2296	5103	1944
E04	1948	2286	2754	1948	2283	2744	5389	1959
E05	81	156	588	80	152	577	2268	757
E06	1993	3045	3585	1988	3028	3540	6336	2227
E07	2839	3487	4198	2830	3474	4173	8801	3171
E08	2435	3015	3554	2435	3003	3529	6787	2414
E09	497	592	686	497	589	679	1430	472
E10	34	68	177	33	67	175	6265	2484
E11	467	1132	1439	466	1130	1435	3303	1112
E12	40	66	106	40	66	105	5877	2397
Total	10773	15258	21772	10756	15202	21633	64320	23635

### Genetic Linkage Map

**FIGURE 2:**
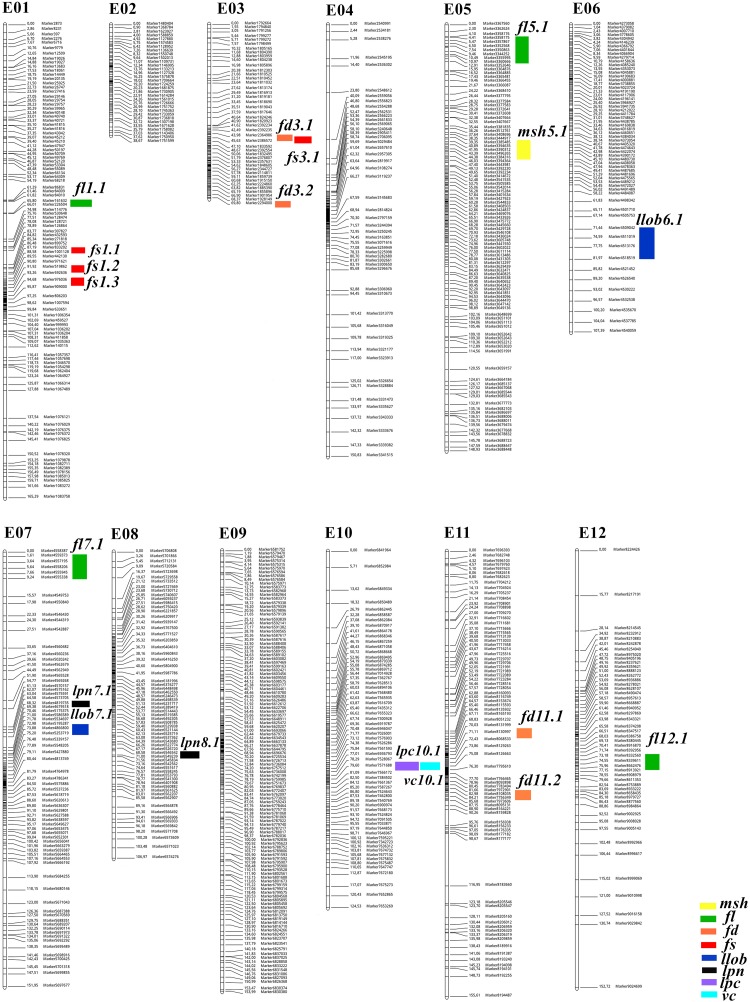
SNP-based genetic linkage map of the interspecific F_2_ population showing positions of QTLs. The numbers to the right of each LG indicate genetic distance (cM) between adjacent markers. The color bars refer to QTLs detected for the eight traits.

After further marker filtering and quality screening, a total of 2,122 SNP markers that met the quality standards were used for genetic map construction. The average depths of the SNPs for female, male, and the offspring were 15.03-fold, 14.09-fold, and 24.68-fold, respectively. JoinMap 5.0 assigned all of the 2,122 markers to 12 eggplant LGs (Figure 2), details of this SNP-based genetic map are presented in Supplementary Table S1 and summarized in Table 2. The total genetic length of the eggplant linkage map was 1530.75 cM with an average marker distance of 0.72 cM. The number of SNP markers in each LG ranged from 90 (LG2) to 273 (LG9), with genetic distances spanning 38.67 cM (LG2) to 165.29 cM (LG1), and mean marker intervals ranged from 0.43 to 1.4 cM. The longest linkage group is LG1, which contains 167 SNPs, whereas the shortest is LG2, containing 90 SNPs.

**TABLE 2:** Information on the SNP-based genetic map of eggplant.

Linkage group ID	Marker no.	Total distance (cM)	Average distance (cM)
E01	167	165.29	0.99
E02	90	38.67	0.43
E03	164	73.89	0.45
E04	206	150.83	0.73
E05	219	148.93	0.68
E06	138	107.39	0.78
E07	166	151.95	0.92
E08	224	106.97	0.48
E09	273	153.97	0.56
E10	147	124.53	0.85
E11	219	155.61	0.71
E12	109	152.72	1.40
Total	2122	1530.75	0.72

### Phenotypic Evaluation

In the present study, the cultivated eggplant “1836” and the wild *S. linnaeanum* “1809” (Figure 1) were used to develop F_2_ segregating populations for QTL analysis of multiple traits. Phenotypic data (including family means, standard errors, and distribution) of eight traits, i.e., main stem height (MSH), fruit length (FL), fruit diameter (FD), fruit shape (FS), leaf lobing (LLOB), leaf prickle number (LPN), leaf prickle color (LPC), and vein color (VC), are presented in Supplementary Table S2. All traits were measured in summer 2018. MSH, FL, FD, and FS could easily be measured or calculated using a ruler or vernier caliper; LLOB and LPN were scored on a 1-7 scale to describe the degree of lobing or number, respectively. Skewness and kurtosis tests showed that all these traits were normally distributed (Supplementary Table S2). LPC ranged in a spectrum from light green to dark purple in the segregating populations and was thus assessed on a 1-4 color scale. Likewise, VC was assessed on a 1-3 scale. They were also treated as quantitative traits. We calculated correlations among all eight traits (Supplementary Table S2). The results showed that LPC and VC had a notably high correlation and that FL was correlated closely with FD and FS. Moreover, considerable correlation was also observed between LLOB and LPN, with a correlation coefficient of 0.65. The highly correlated traits may share some tightly linked markers and/or candidate genes, such as the prickles on leaf and stem, fruit length and diameter, and the color of leaf vein and prickle. In fact, the genes involved in anthocyanin accumulation may simultaneously affect the colors of leaf vein, stem, prickle, and even fruit epicarp. This is useful in candidate gene prediction and function analysis. Fruit shape is determined by length and diameter; thus, breeding for eggplant fruit shape should take both into consideration.

### QTL Mapping of Morphological Traits in Eggplant

A total of 19 QTLs for main stem height (*msh*), fruit length (*fl*), fruit diameter (*fd*), fruit shape (*fs*), leaf lobing (*llob*), leaf prickle number (*lpn*), leaf prickle color (*lpc*), and vein color (*vc*) were identified in the F_2_ eggplant population (Table 3). The phenotypic variance explained (PVE) by the 19 QTLs ranged from 4.08-55.23%, and LOD values ranged from 2.09-28.75. The number of SNP markers within each QTL varied from 2 to 11, and the genetic distance interval of the QTLs ranged from 0.15 to 10.53 cM. The physical locations of these QTLs on eggplant chromosomes were obtained by BLAST marker sequences with the eggplant reference genome. The QTLs were distributed on nine chromosomes/linkage groups: LG1, 3, 5, 6, 7, 8, 10, 11, and 12 (Figure 2). Detailed sequence information and alignment positions for all markers are presented in Table 3 and Supplementary Table S3. The SNPs of each QTL were mapped to the eggplant genome (Barchi et al., 2019) to anchor the physical locations, and the distribution of these QTL loci on chromosomes determined with two terminal markers are shown in Supplementary Figure S1.

**TABLE 3:** Detailed information on QTLs detected for the eight traits in the interspecific F_2_ population.

		Linkage	SNP	Linkage map	Interval	Genome	Interval				
No.	QTL	group ID	no.	position (cM)	size (cM)	position (Mb)	size (Mb)	LOD	ADD	DOM	PVE
1	*msh5.1*	E05	11	39.35–44.38	5.03	5.98–7.93	1.95	2.29	3.08	2.15	6.80
						13.57–19.22	5.65				
2	*fl1.1*	E01	2	65.80–66.01	0.21	28.69–35.85	7.16	3.44	–3.09	–5.84	12.00
3	*fl5.1*	E05	11	4.10–11.96	7.85	0.21–2.71	2.5	2.27	5.39	–0.76	13.06
4	*fl7.1*	E07	5	1.61–9.24	7.63	2.38–3.08	0.7	2.28	2.16	–3.29	4.08
5	*fl12.1*	E12	3	74.02–74.55	0.53	18.05–31.63	13.58	3.77	–4.72	8.94	24.05
6	*fd3.1*	E03	2	44.28–44.43	0.15	4.46–4.97	0.51	2.47	–1.31	–3.66	11.98
7	*fd3.2*	E03	3	69.81–70.40	0.60	60.33–66.04	5.71	2.36	–0.60	–3.38	8.50
8	*fd11.1*	E11	4	71.47–71.87	0.40	58.64–59.84	1.2	2.37	0.63	3.28	7.95
9	*fd11.2*	E11	4	82.29–82.98	0.68	13.15–28.19	15.04	2.09	0.49	3.04	6.62
						55.83–58.83	0				
10	*fs1.1*	E01	2	87.87–88.58	0.71	110.94–124.29	13.35	3.18	0.09	–0.14	6.92
11	*fs1.2*	E01	4	91.93–92.46	0.54	70.35–72.64	2.29	2.75	0.11	–0.15	8.71
12	*fs1.3*	E01	3	94.23–94.47	0.24	111.30–111.91	0.61	3.16	0.12	–0.16	10.92
13	*fs3.1*	E03	2	44.98–45.32	0.34	73.35–73.74	0.39	2.96	–0.14	–0.08	10.76
14	*llob6.1*	E06	4	71.44–81.97	10.53	104.00–105.00	1	8.99	1.45	–0.15	55.23
15	*llob7.1*	E07	3	73.48–74.35	0.87	81.94–87.96	6.02	2.63	0.61	0.00	9.68
16	*lpn7.1*	E07	2	68.32–69.38	1.06	17.29–33.55	16.26	2.42	0.65	0.24	9.69
17	*lpn8.1*	E08	4	68.58–71.00	2.42	103.00–106.00	3	2.44	–0.60	–0.17	7.39
18	*lpc10.1*	E10	9	79.02–79.94	0.92	87.85–95.97	8.12	27.65	–0.53	1.49	36.95
19	*vc10.1*	E10	8	79.44–80.14	0.70	85.12–95.97	10.85	28.75	–0.68	0.66	52.10

One QTL for MSH, designated *msh5.1*, was detected on LG5 and could explain 6.8% of the observed phenotypic variation. Eleven SNPs were uncovered within this QTL region. However, although QTL *msh5.1* on the genetic map is a continuous interval, it corresponded to two physical regions on chromosome 5: 5.98-7.93 Mb and 13.57-19.22 Mb.

Four QTL loci, designated *fl1.1*, *fl5.1*, *fl7.1*, and *fl12.1*, were detected for FL and were located on LG1, 5, 7, and 12, respectively. The most prominent QTL, *fl12.1*, explained 24.05% of the phenotypic variation, followed by *fl1.1* and *fl5.1*, which explained 12.00 and 13.06% of the fruit length variation, respectively. The lowest contribution rate was 4.08% for QTL *fl7.1*. In addition, up to 11 SNPs were identified within the QTL region of *fl5.1*. Among the four QTLs, except that Marker8964317 of *fl12.1* is far from the other two markers, all other markers are within a reasonable genome position (Table 3 and Supplementary Table S3). For *fl12.1*, the three markers within the region were positioned apart on chromosome 12, at 18.05, 31.63, and 90.73 Mb; the interval 18.05-31.63 Mb were retained since the other site, 90.73 Mb, was far from the other two sites.

For fruit diameter, four QTLs were detected on linkage groups 3 and 11, two for each LG. The highest contribution rate was 11.98% for *fd3.1*, followed by *fd3.2* (8.50%), *fd11.1* (7.95%), and *fd11.2* (6.62%). The genetic interval of each QTL ranged from 0.15 to 0.68 cM. The case in *fl12.1* was also observed for *fd3.2. fd11.1* and *fd11.2* spanned 71.47-71.87 cM and 82.29-82.98 cM on linkage group 11, respectively. Among the four SNP markers corresponding to *fd11.2*, Marker8108035 and Marker8108041 were at the same position of 55.83 Mb whereas the other two markers covered an interval of 13.15-28.19 Mb.

Four QTLs were detected for the FS index, with the contribution rates ranging from 6.92% (*fs1.1*) to 10.92% (*fs3.1*). There were three QTLs on LG1 (*fs1.1*, *fs1.2*, *fs1.3*), and the genetic locations were relatively close together. The chromosomal locations compared with *fs1.3* (111.30-111.91 Mb) were within the chromosomal location region of *fs1.3* (110.94-124.29 Mb). The other QTL was located on LG3, and its position was close to that of *fd3.1*.

The LLOB was controlled by two QTLs, *llob6.1* and *llob7.1* on LG6 and LG7, respectively. The QTL *llob6.1* had a major effect on eggplant leaf division, explaining 55.23% of the phenotypic variation with an LOD threshold of 8.99. For *llob7.1*, the physical locations of the three markers were 81.94-87.96 Mb and 38.09 Mb. For LPN, two QTL loci were located on LG7 and LG8, *lpn7.1* and *lpn8.1*, with contribution rates of 9.69 and 7.39%, respectively. The physical region of *lpn7.1* was 17.29-33.55 Mb.

One major-effect QTL (*lpc10.1*) detected for LPC was located on LG10 and explained 36.95% of the phenotypic variation, with an LOD score of 27.65. The QTL for leaf vein color, *lvc10.1*, was also on LG10, and had an overlapped genetic region with *lpc10.1*. The leaf vein color variation explained by *vc10.1* was 52.10%.

## Discussion

### The Mapping Populations in Eggplant

Eggplant linkage maps were constructed with both intra- and inter-specific populations (Nunome et al., 2001, 2003; Doganlar et al., 2002a; Wu et al., 2009; Barchi et al., 2010, 2012, 2018; Miyatake et al., 2012, 2016; Lebeau et al., 2013; Portis et al., 2014). The intraspecific genetic map generated using an F_2_ population derived from 305E40 × 67/3 has wide application. Researchers managed to reuse the F_2_ materials by cutting and grafting the established vegetative cuttings and then obtained phenotypic data (Barchi et al., 2010, 2012, 2018; Portis et al., 2014; Toppino et al., 2016). This population was used to identify QTLs underlying anthocyanin pigmentation, early yield, fruit-related traits, and resistance to *Fusarium* and *Verticillium*. However, the linkage maps derived from 305E40 × 67/3 contained only ∼400 markers.

Similar to what has been observed in other domesticated crops, artificial selection has caused a dramatic reduction in genetic variation in the cultivated eggplant germplasm, resulting in limited polymorphisms within intraspecific populations and reduced resistance (Daunay et al., 1991; Rotino et al., 2014). Wild Solanum species, which include *S. linnaeanum*, *S. aculeatissimum*, *S. sisymbrifolium*, and *S. torvum*, represent valuable reservoirs of potentially useful resistant alleles for eggplant breeding (Collonnier et al., 2001; Frary et al., 2003; Daunay, 2008; Liu et al., 2015). *S. linnaeanum* is closely related to *S. melongena* (Mace et al., 1999) and was reported to exhibit resistance to Verticillium wilt, black root rot, potato virus, and salinity (Daunay et al., 1991). Fertile F_1_ hybrids were only obtained from a cross between *S. linnaeanum* and *S. melongena* (Collonnier et al., 2001). Doganlar et al. (2002a) constructed the first interspecific genetic map with 334 points in eggplant by crossing *S. linnaeanum* MM195 with *S. melongena* MM738. Since then, this interspecific population has been widely used in QTL mapping for fruit, flower, and leaf characteristics, as well as comparative studies with tomato (Doganlar et al., 2002a, b; Frary et al., 2003, 2014; Wu et al., 2009). The genetic map with increased resolution constructed by Frary et al. (2014) reached 735 markers. Nevertheless, the population size of *S. linnaeanum* MM195 × *S. melongena* MM738 was relatively small, containing only 58 individuals.

In the present study, we performed interspecific hybridization between eggplant cultivar “1836” and the wild relative *S. linnaeanum* “1809.” An F_1_ hybrid was successfully generated and was then self-pollinated to produce an F_2_ population with 121 individuals. However, some of the F_2_ plants could not bear fruits due to their interspecific nature. The enlarged interspecific population provided raw materials for the introgression of valuable traits from wild species into eggplant cultivars and the identification of QTLs controlling domestication-related traits.

### Construction of a Saturated SNP-Based Genetic Map Using SLAF-Seq

To date, over twenty linkage maps have been constructed for eggplant using various types of molecular markers, including RAPDs, AFLPs, and conserved ortholog set (II) (COS/COSII) markers, RFLPs, and SSRs (Table 4). Most of them are not saturated due to the low frequency of DNA polymorphism. In the present study, we developed a high-density SNP-based genetic linkage map in eggplant using SLAF sequencing.

**TABLE 4:** Summery of previously reported genetic maps in eggplant.

				Total				
	Interspecific or	Group	Population	Linkage	genetic	Marker		
Cross parent	intraspecific	type	size	group no.	distance	no.	Marker type	References
EPL-1 × WCGR112-8	Intraspecific	F_2_	168	21	779.2	181	RAPD, AFLP	Nunome et al., 2001
*Solanum linnaeanum* MM195 × *S. melongena* MM738	Interspecific	F_2_	58	12	1480	334	tomato cDNA, genomic DNA, COS, RFLP	Doganlar et al., 2002a
*S. linnaeanum* MM195 × *S. melongena* MM738	Interspecific	F_2_	58	12		207	RFLP	Doganlar et al., 2002b
*S. linnaeanum* MM195 × *S.melongena* MM738	Interspecific	F_2_	58	12		207	RFLP	Frary et al., 2003
EPL-1 × WCGR112-8	Intraspecific	F_2_	168	17	716.9	162	RAPD, AFLP, SSR	Nunome et al., 2003
*S. sodomeum*(=*S. linneanum*) × *S. melongena* Buia	Interspecific	F_2_	48	13	736	273	RAPD, AFLP	Sunseri et al., 2003
EPL-1 × WCGR112-8	Intraspecific	F_2_	94	14	959.1	236	SSR	Nunome et al., 2009
*S. linnaeanum* MM195 × *S. melongena* MM738	Interspecific	F_2_	58	12	1535	347	COSII	Wu et al., 2009
305E40 × 67/3	Intraspecific	F_2_	141	12	718.7	238	AFLP, SSR, RFLP, Rfo-sa1 CAPS	Barchi et al., 2010
305E40 × 67/3	Intraspecific	F_2_	156	12	1389.7	415	SNP, SSR, COS	Barchi et al., 2012
LS1934 × WCGR112-8, AE-P03 × LS1934	Intraspecific	F_2_	90,93	12	1285.5	952	SSR, SNP	Fukuoka et al., 2012
AE-P03 × LS1934	Intraspecific	F_2_	135	12	1414.6	250	SSR, SNP SSR, SNP	Miyatake et al., 2012
Nakate-Shinkuro × AE-P03	Intraspecific	F_2_	93	12	1153.8	174		
MM738 × AG91-25	Intraspecific	F_6_	178	18	884	119	AFLP, SSR, SRAP	Lebeau et al., 2013
*S. linnaeanum* MM195 × *S. melongena* MM738	Interspecific	F_2_	58	12		736	AFLP, RFLP, COSII	Frary et al., 2014
305E40 × 67/3	Intraspecific	F_2_	156	12	1389.7	415	SNP, SSR, COS	Portis et al., 2014
LS1934 × WCGR112-8, EPL-1 × WCGR112-8	Intraspecific	F_2_	90,120	12	1280.6	1745	SNP, SSR	Hirakawa et al., 2014
LS1934 × WCGR112-8	Intraspecific	F_2_	90	12	1280.6	1193	SNP,	Miyatake et al., 2016
EPL-1 × WCGR112-8	Intraspecific	F_2_	120	12	1280.6	602	SSR	
AE-P03 × LS1934	Intraspecific	F_2_	93	12	1285.5	952		
305E40 × 67/3	Intraspecific	F_2_	156	12	1389.7	415	SNP, SSR, COS	Toppino et al., 2016
305E40 × 67/3	Intraspecific	F_2_	156	12	1390	418	SNP, SSR, COS, HRM	Barchi et al., 2018

The first genetic map in eggplant contained 181 markers, all of which were dominant markers (RAPDs and AFLPs) that were sorted into 21 linkage groups (Nunome et al., 2001). After that, several linkage maps were constructed using mostly dominant markers (Doganlar et al., 2002a, b; Barchi et al., 2010), although the co-dominant SSR markers were introduced into map construction (Nunome et al., 2003). Nunome et al. (2009) developed an enriched SSR-based genetic map containing 236 SSR markers, which were assigned into 14 linkage groups spanning 959.1 cM, with a mean marker interval of 4.3 cM. However, the marker density was still far from saturated. SNPs are the most abundant and stable form of genetic variation in most plant genomes, which have outstanding advantages for the construction of saturated genetic maps. Fukuoka et al. (2012) constructed an integrated linkage map using two mapping populations that include 952 markers (313 SSRs and 623 SNPs) spanning 1285.5 cM. In 2014, another integrated map was constructed with 1745 markers to facilitate eggplant genome assembly, which includes 547 SNPs and 221 SSRs, spanning 1280.6 cM (Hirakawa et al., 2014). Although the marker number was improved in the two integration maps, the applications in QTL mapping were rather limited.

SLAF sequencing takes advantage of high-throughput sequencing and genotyping, providing a powerful tool for genome-wide SNP discovery and marker development. In the present study, we conducted large-scale SNP screening, and 13,455,526 SNP markers were developed, from which 11,386,169 SNPs were successfully encoded. However, a considerable amount of the SNPs were segregation distorted in the interspecific F_2_ population. Thus, we performed a strict marker filtering process before map construction. Finally, a saturated genetic map with 2,122 high-quality SNP markers was constructed using SLAF-seq (Table 2 and Figure 2), which is a significant improvement in marker number as compared to the individual maps (Frary et al., 2014; Miyatake et al., 2016; Barchi et al., 2018) as well as the two integrated linkage maps (Fukuoka et al., 2012; Hirakawa et al., 2014). The total genetic length of the SNP-based linkage map was 1530.75 cM, and the average marker distance was narrowed down to 0.72 cM. The marker number in each LG ranged from 90 to 273 SNPs, with mean marker intervals ranging from 0.43 cM to 1.4 cM. This high-density genetic map establishes a foundation for accurate and reliable mapping of QTLs, as well as the identification of candidate genes underlying important traits in eggplant.

### QTL Mapping of Morphological Traits in Eggplant

Eggplants exhibit wide biodiversity among local landraces and wild relatives, with considerable variations in fruit size and color, leaf morphology, and pathogen resistance. Unlike most of the other major Solanaceous crops, which are native to the New World, eggplant has a unique phylogeny of Old World domestication that occurred in India and Southern China (Fukuoka et al., 2010; Meyer et al., 2012; Cericola et al., 2013; Albert and Chang, 2014). The wild forms of eggplant are usually pricky with small, bitter fruits; however, selection during domestication resulted in elongated and palatable fruits with fewer prickles in cultivated eggplant (Choudhury, 1995). The two parental lines used in the present study, *S. melongena* “1836” and the wild *S. linnaeanum* “1809,” have contrasting phenotypes (Figure 1), making them valuable for investigating the molecular mechanisms underlying domestication-related traits.

Using the high-density SNP map and the interspecific F_2_ population, we identified a total of 19 QTLs for main stem length and fruit and leaf morphology (Table 3 and Figure 2). While no QTL loci were detected on three LGs (2, 4, and 9), all of the other nine LGs had QTL distributions. The phenotypic variance explained by the QTLs ranged between 4.08 and 55.23%, and the genetic distance interval varied from 0.15 to 10.53 cM. We detected one QTL for main stem height (*msh5.1*) on LG5, explaining 6.8% of the phenotypic variation. The genetic interval was 5.03 cM, covering 11 SNPs. Previous reports on QTLs for eggplant fruit and leaf traits are rather limited; we summarize previously mapped QTLs related to the eight traits in the present study in Supplementary Table S3. Doganlar et al. (2002b) identified three QTLs for eggplant fruit length using the F_2_ population derived from *S. linnaeanum* “MM195” × *S. melongena* “MM738” and RFLP markers; the three QTLs (i.e., *fl2.1*, *fl9.1*, and *fl11.1*) accounted for 23-29% of the fruit length variation. Using the same population and a 736-point genetic map, Frary et al. (2014) detected five QTLs impacting fruit length that were distributed on LG2, 7, and 9 (*fl1.1*, *fl2.1*, *fl2.2*, *fl7.1*, and *fl9.1*). Another report used an intraspecific population derived from 305E40 × 67/3 and a genetic map with 415 markers, and six QTLs affecting fruit length were detected over six LGs: LG1, 2, 3, 7, 8, and 11 (Portis et al., 2014). In the present study, four QTLs (*fl1.1*, *fl5.1*, *fl7.1*, and *fl12.1*) were identified for fruit length, and the QTL *fl12.1* explained 24.05% of the variation for fruit length. Thus, fruit length-related QTLs in eggplant are distributed on nine different LGs.

For fruit diameter, we detected four QTLs on LG3 (*fd3.1*, *fd3.2*) and LG11 (*fd11.1, fd11.2*), with the genetic interval of each QTL ranging between 0.15 and 0.68 cM. These QTLs accounted for 6.62 to 11.98% of the observed phenotypic variation. Doganlar et al. (2002b) identified two QTLs on LG1 (*fd1.1*) and LG11 (*fd11.1*), which explained 17% of the total variation for FD. Whereas Portis et al. (2014) adopted three FD parameters for the intraspecific population (i.e., fd1/2, fd3/4, and fdmax), three to seven QTLs were mapped on LG2, 3, 4, 7, 11, and 12. FS-related QTLs were also detected in the two aforementioned studies, which were distributed on LG1, 2, 3, 7, and 11. In the present study, we identify four QTLs for fruit shape index, among which three were on LG1 (*fs1.1*, *fs1.2*, *fs1.3*) and one on LG3 (*fs3.1*). The genetic locations of *fs1.1*, *fs1.2*, and *fs1.3* were very close together, suggesting that they may function as a single locus. Collectively, the three QTLs explained 26.55% of the fruit shape variation.

In total, six QTLs were detected for leaf morphology-related traits in this study. LPC and LVC were highly correlated (Supplementary Table S2), and as expected, *vc10.1* had an overlapping genetic location with *lpc10.1*. *llob6.1* was identified as a major-effect QTL that accounted for 55.23% of the phenotypic variation. In previous studies, Frary et al. (2003) identified two QTLs for leaf lobing, on LG6 (*llob6.1*) and LG10 (*llob10.1*); after increasing the marker density on the original map, Frary et al. (2014) identified four QTLs on LG5, 6, and 7 using the same population. In the previous studies, each QTL interval was only covered by one or two markers (Supplementary Table S3); with the 2,122-point SNP-based map, up to 11 SNPs were harbored in a single QTL locus in this study. This increase in mapped markers could better facilitate the fine mapping of these QTLs in further analysis.

**FIGURE 3:**
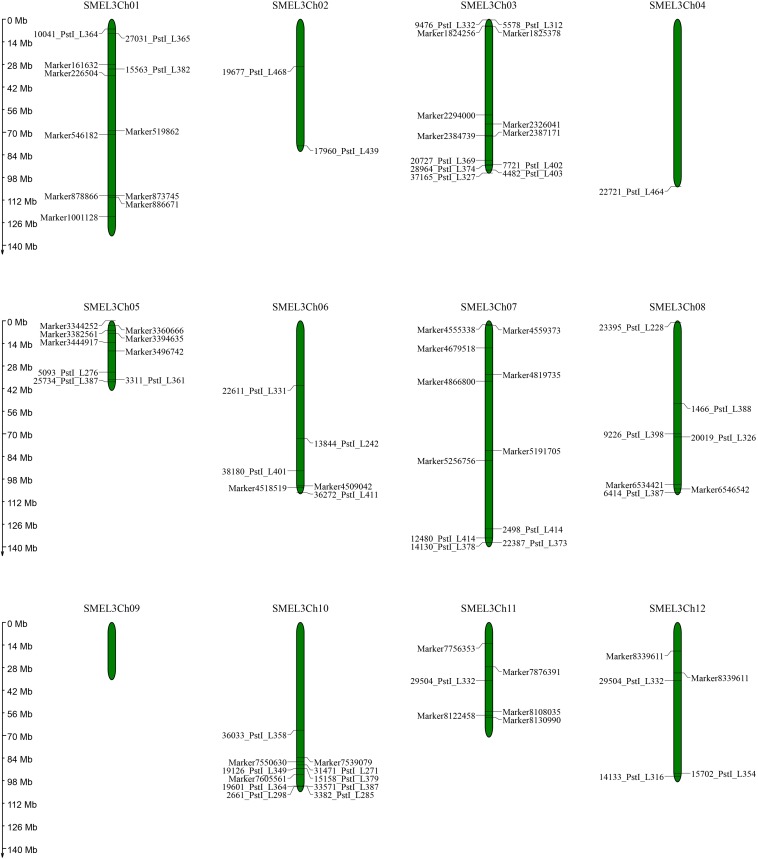
Graphic view of the distribution of markers associated with related traits on eggplant chromosomes.

To better demonstrate the mechanisms underlying fruit and leaf morphology traits in eggplant, we performed comparative analysis between the QTLs in the present study and previous QTL analysis and association studies (Barchi et al., 2012; Cericola et al., 2014; Portis et al., 2014, 2015) based on marker sequences and the eggplant reference genome (Barchi et al., 2019). A graphic view of the distribution of the QTLs and markers associated with related traits on eggplant chromosomes was produced (Figure 3 and Supplementary Table S3). There were no relevant markers or QTL loci on chromosome 9, whereas all of the other 11 chromosomes had marker distribution. The SNPs in the QTLs we located could be associated with some of the markers in previous studies. For example, markers 19126_PstI_L349, 31471_PstI_L271, and 15158_PstI_L379 were shown to be related to the anthocyanin content of leaf veins (Portis et al., 2014), and these markers are also located in *lpc10.1* and *vc10.1* in the present study. Thus, we speculated that there were candidate genes related to anthocyanin accumulation in leaves in this region. Marker 29504_PstI_L332, which has been related to fruit length, diameter, and fruit shape (Portis et al., 2014), is close to *fl12.1* in the present study. Nonetheless, there are also inconsistencies among different studies. The QTL controlling fruit diameter determined by markers 9476_PstI_L332 and 5578_PstI_L312 was anchored to the eggplant chromosome 3, close to the QTL *fd3.1* we detected. In addition, marker 36272_PstI_L411 was related to the leaf prickle number trait but close to the QTL of leaf lobing we located. Notably, some markers are relatively far apart on the chromosome in terms of physical location, whereas they were close on the genetic map. This is likely due to errors in either linkage distance calculation or misassembly of the eggplant genome. Another possible reason is that the two parents of the mapping population were different from the genome sequencing eggplant material, especially the wild species *S. linnaeanum*, resulting in additional genetic polymorphism.

## Conclusion

In conclusion, the high-density SNP-based genetic map and QTLs controlling agronomic traits for eggplant in the present study provide an important foundation for developing tightly linked markers for marker-assisted breeding, as well as fine mapping and gene mining of related traits, especially the QTLs presented in the interspecific population, which could facilitate the demonstration of eggplant domestication. We also assigned the QTLs to eggplant chromosomes and have provided the physical positions of the markers and their sequences. QTL loci of the same traits in multiple studies should be anchored to the same high-quality eggplant genome; the hotspots controlling those traits could then be determined based on repeatability, and further precise predictions could be made of the candidate genes for functional analysis. However, more marker sequence information corresponding to the QTLs needs to be disclosed.

## Data Availability Statement

Raw sequence reads have been submitted to the NCBI Sequence Read Archive under the accession number PRJNA577305. The datasets supporting the conclusions drawn in this study are included within the manuscript and the Supplementary Tables.

## Author Contributions

QW conceived and designed the experiments, performed the experiments, analyzed the data, prepared figures and/or tables, authored or reviewed drafts of the manuscript, and approved the final draft. WW analyzed the data, authored or reviewed drafts of the manuscript, and approved the final draft. TH, HH, and JW analyzed the data, contributed reagents, materials, and analysis tools, authored or reviewed drafts of the manuscript, and approved the final draft. CB conceived and designed the experiments, authored or reviewed drafts of the manuscript, and approved the final draft.

## Conflict of Interest

The authors declare that the research was conducted in the absence of any commercial or financial relationships that could be construed as a potential conflict of interest.

## Footnotes

1 http://faostat3.fao.org

2 https://www.kyazma.nl/index.php/JoinMap/

3 http://www.rqtl.org/

## Supplementary Material

FIGURE S1 Distribution of the QTLs on eggplant chromosomes.

TABLE S1 Genotyping results of the markers and detailed information of the SNP-based genetic linkage map of eggplant.

TABLE S2 Phenotypic data and ANOVA analysis of the interspecific F_2_ population.

TABLE S3 Summary of the previously reported QTLs related to fruit and leaf traits of eggplant.
