# Supplementary figures and images for: Construction of a SNP-Based Genetic Map Using SLAF-Seq and QTL Analysis of Morphological Traits in Eggplant
Source: Front Genet. 2020 Mar 11;11:178. doi: 10.3389/fgene.2020.00178 (PMC7078336; doi:10.3389/fgene.2020.00178)

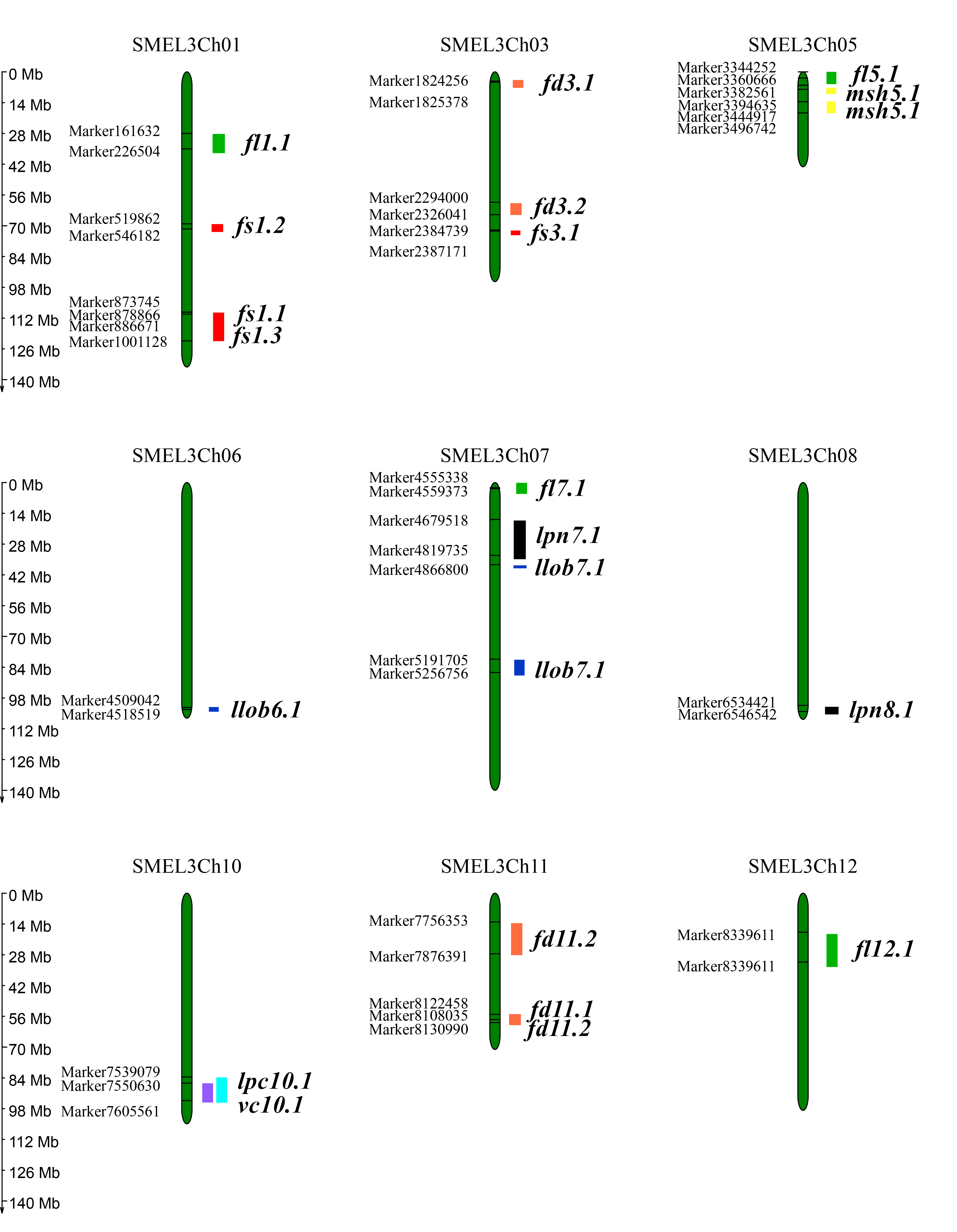

Supplement: FIGURE S1 — Distribution of the QTLs on eggplant chromosomes. [file Image_1.JPEG]
